# Developmental trajectories of child and adolescent emotional problems: associations with early adult alcohol use behaviors

**DOI:** 10.1111/jcpp.14034

**Published:** 2024-06-26

**Authors:** Tong Chen, Olakunle A. Oginni, Laurie J. Hannigan, Thalia C. Eley, Jennifer L. Maggs, Ashley N. Linden‐Carmichael, Jenae M. Neiderhiser

**Affiliations:** ^1^ Department of Psychology The Pennsylvania State University University Park PA USA; ^2^ The Social, Genetic and Developmental Psychiatry Centre, Institute of Psychiatry, Psychology and Neuroscience King's College London London UK; ^3^ Department of Mental Health Obafemi Awolowo University Ile‐Ife Nigeria; ^4^ Nic Waals Institute Lovisenberg Diakonale Hospital Oslo Norway; ^5^ Department of Mental Disorders Norwegian Institute of Public Health Oslo Norway; ^6^ MRC Integrative Epidemiology Unit at the University of Bristol Bristol UK; ^7^ Human Development and Family Studies The Pennsylvania State University University Park PA USA; ^8^ Edna Bennett Pierce Prevention Research Center The Pennsylvania State University University Park PA USA

**Keywords:** Behavior problems, alcohol abuse, longitudinal studies, development, genetics

## Abstract

**Background:**

Whether emotional problems during childhood and adolescence are longitudinally associated with adult alcohol use behaviors is unclear. This study examined associations between developmental trajectories of emotional problems and early adult alcohol use behaviors, while considering co‐occurring conduct problems, developmental change/timing, sex differences, and potential confounds.

**Methods:**

Participants were from the Twins Early Development Study (analytic *N* = 19,908 individuals). Emotional and conduct problems were measured by parent reports at child ages 4, 7, and 9 years and via self‐reports at ages 9, 11, and 16 years on the Strengths and Difficulties Questionnaire. Alcohol use behaviors (alcohol consumption and alcohol‐related problems) were self‐reported by the twins on the Alcohol Use Disorders Identification Test at age 22 years. Piecewise latent growth curve models described nonlinear developmental trajectories of emotional and conduct problems from ages 4 to 16. At age 22, alcohol use was regressed on emotional and conduct problems' intercepts and slopes from piecewise latent growth curve model and sex differences in regression coefficients were tested. Using twin modeling, Cholesky decompositions and direct path models were compared to test whether significant phenotypic associations were best explained by direct phenotypic influences or correlated genetic and environmental influences.

**Results:**

Emotional problems had different associations with alcohol‐related problems versus alcohol consumption. After accounting for direct influences from conduct problems, emotional problems were not associated with *alcohol‐related problems*, while emotional problems at age 9 were negatively associated with *alcohol consumption* in males.

**Conclusions:**

Overall, findings did not support emotional problems as prospective risk factors for severe alcohol use above and beyond risks associated with conduct problems. Sex‐ and age‐specific links between emotional problems and alcohol consumption in early adulthood may be worthy of further exploration, particularly as twin analyses improved our confidence that such links may be underpinned by causal mechanisms.

While it has been theorized that emotional problems during childhood and adolescence can be associated with alcohol use behaviors in adolescence and adulthood (e.g. Zucker, 2006; Zucker, Donovan, Masten, Mattson, & Moss, 2008), empirical evidence has been mixed (see Hussong, Ennett, Cox, & Haroon, 2017; Ning, Gondek, Patalay, & Ploubidis, 2020 for systematic reviews). One reason for mixed findings could be the effects of co‐occurring externalizing problems. Studies that did not control for externalizing problems often reported associations between emotional problems and more alcohol use (Berg et al., 2019; McKenzie et al., 2011; Pesola, Shelton, & van den Bree, 2014). However, when externalizing problems were included as a covariate, these associations were attenuated or reversed (e.g. Foster, Hicks, & Zucker, 2018; Maggs, Patrick, & Feinstein, 2008; Scalco et al., 2021). Such findings suggest that when accounting for risks explained through externalizing problems, emotional problems may be uniquely associated with less alcohol use or not associated at all.

Another reason for the mixed findings could be the lack of focus on the developmental nature of emotional problems. Levels of emotional problems change across childhood and adolescence, and individuals with the same initial level of emotional problems at any time may follow different trajectories thereafter (e.g. Dekker et al., 2007; Hannigan et al., 2018; Nivard et al., 2017). These different trajectories may in turn lead to different alcohol use behaviors. A few studies have shown that both the initial level of emotional problems and an increase in emotional problems during adolescence were associated with more alcohol use in adolescence or adulthood (Edwards et al., 2014; Leve, Harold, Van Ryzin, Elam, & Chamberlain, 2012). However, these studies focused solely on emotional problems during adolescence and did not include earlier developmental periods such as childhood.

Notably, changes in emotional problems across childhood and adolescence are often nonlinear, suggesting that the rates of changes may not be consistent across development (e.g. Cohen, Andrews, Davis, & Rudolph, 2018; Dekker et al., 2007; Hannigan et al., 2018; Nelemans, Hale, Branje, Meeus, & Rudolph, 2018; Nivard et al., 2017). Therefore, the developmental trajectory of emotional problems should be modeled across multiple developmental periods to capture the full picture. Furthermore, previous studies suggested that emotional problems that occur in different developmental periods may differentially influence later alcohol use behaviors. In a systematic review, Ning et al. (2020) reported alcohol use in adulthood was negatively associated with anxiety in *early* adolescence, positively associated with anxiety across the *entire* adolescent period, and not associated with anxiety in childhood at all. No conclusions about potential developmental timing effects for depression could be made because most studies measured depression only during adolescence (Ning et al., 2020). Given these findings, modeling developmental trajectories of emotional problems across the entire childhood and adolescence period can provide informative insights into the effects of emotional problems on adult alcohol use.

Additionally, sex differences have been reported in associations between developmental trajectories of emotional problems and alcohol use behaviors. Increases in emotional problems during adolescence were associated with more alcohol use in females but not in males (Edwards et al., 2014; Leve et al., 2012). This is somewhat inconsistent with the broader literature, in which no consistent evidence of sex differences in associations between emotional problems and alcohol use behaviors has been found (e.g. Edwards, Gardner, Hickman, & Kendler, 2016; Pesola et al., 2014; Soloski, 2020). Therefore, more studies testing sex differences in associations between developmental trajectories of emotional problems and alcohol use behaviors are needed to provide more robust evidence.

Finally, although there has been extant research examining longitudinal associations between emotional problems and alcohol use, incorporating genetically informed designs in these investigations may be especially valuable because it allows strengthened causal inference in the relationship between emotional problems and later use of alcohol. It is possible that significant associations between emotional problems and alcohol use behaviors may be confounded by shared genetic and/or environmental factors (Tully & Iacono, 2016). In support of this possibility, studies that controlled for genetic and environmental influences shared among family members found that associations between emotional problems and alcohol use behaviors were eliminated or attenuated (Foster, 2019; Sihvola et al., 2008; Stephenson et al., 2020; Virtanen et al., 2021). In the present study, we used twin data to strengthen causal inference by comparing models that assumed direct phenotypic influences from emotional problems to alcohol use versus models that specified correlated genetic and environmental influences. A better fit of the former model would provide more confidence that significant associations between emotional problems and alcohol use (if observed) may be due to causal mechanisms.

To address the above‐mentioned gaps in the literature using a genetically sensitive twin design, the present study had three main aims. First, to examine whether developmental trajectories of emotional problems across childhood and adolescence were uniquely associated with early adult alcohol use behaviors when controlling for developmental trajectories of conduct problems (externalizing problems focused on delinquency and aggressive behaviors). Second, to examine whether sex differences existed in associations described in Aim 1. Third, to examine whether emotional problems had direct influences on alcohol use, or that associations between emotional problems and alcohol use were best explained by correlated genetic and/or environmental influences. Finally, a post hoc exploratory aim was added to understand whether the effects of emotional problems differed for alcohol consumption versus alcohol‐related problems, which helped address whether our findings have implications for normative or problematic drinking behavior, or both.

## Methods

### Participants

Participants came from the Twins Early Development Study (TEDS; Lockhart et al., 2023), a longitudinal study that follows twins born in England and Wales between 1994 and 1996. In this study, we used data at twin ages 4, 7, 9, 11, 16, and 22 years and background information collected at the initial contact (~18 months). The analytic sample included all TEDS twins with data available on at least one measurement occasion. Participants with serious medical conditions, extreme adverse perinatal conditions, missing essential background variables, or zygosity were excluded from analyses (https://www.teds.ac.uk/datadictionary/exclusions.htm). This resulted in a sample size of 19,908 individuals from 10,325 twin pairs (6,853 monozygotic twin pairs, 6,540 same‐sex dizygotic twin pairs, 6,515 opposite‐sex dizygotic twin pairs, 51% female, 93% white). TEDS was established to be representative of the population in England and Wales, although demographics change with study waves (Lockhart et al., 2023). Attrition analyses were conducted to compare participants with data at age 22 versus those without (Table S1). Participants who provided data at the age 22 wave were more likely to be female, white, had higher family socioeconomic status at the initial contact, and had lower emotional and conduct problems across waves. Informed consent was obtained from all participating parents and adult twins. Ethical approval was received from the King's College London Research Ethics Committee (reference: PNM/09/10‐104).

### Measures

#### Emotional and conduct problems

Emotional and conduct problems were measured by the Strengths and Difficulties Questionnaire (SDQ; Goodman, 1997) at ages 4, 7, 9 by parent reports, and at ages 9, 11, 16 by twin self‐reports. Emotional problems measured whether a child/adolescent was worried, unhappy, nervous/clingy, easily scared, or complained about somatic symptoms. In the age 4, parent report on emotional problems, the ‘nervous or clingy…’ item was missing and was replaced with a proxy item (‘tends to be shy and timid’). In the age 16, self‐report on emotional problems, the ‘I am often unhappy, downhearted or tearful’ was missing and was replaced with a similar item ‘I felt miserable or unhappy’ from the Mood and Feelings Questionnaire (Angold, Costello, Messer, & Pickles, 1995). Conduct problems measured whether a child/adolescent had tantrums, was obedient, fought, lied, and stole things. Both subscales had five items, with a 3‐point rating scale from ‘not true (0)’ to ‘certainly true (2).’ Total scores for emotional/conduct problems were computed as the mean of all available items multiplied by the number of total items (5), ranging from 0 to 10. The SDQ subscales showed acceptable/good validity (Goodman, 2001) and reliability (the ordinal Cronbach's *⍺* ranged from .69 to .82 in this sample across all the timepoints of interest).

#### Alcohol use behaviors

Alcohol use behaviors at age 22 for each twin were measured by the 10‐item Alcohol Use Disorders Identification Test (AUDIT), a self‐reported screening tool for hazardous/harmful alcohol use (Saunders, Aasland, Babor, De la Fuente, & Grant, 1993). Items measured both *alcohol consumption* (e.g. frequency of drinking, number of drinks consumed on a typical day) and *alcohol‐related problems* (e.g. getting injured because of drinking). Each item was scored from 0 to 4, and a total AUDIT score (AUDIT‐Total) was computed as the mean of all available items multiplied by the number of items (10), ranging from 0 to 40 (ordinal Cronbach's *⍺* = .89). The total score could also be separated into two parts, with one score measuring alcohol consumption (calculated from items 1–3; ordinal Cronbach's *⍺* = .68) and one score measuring alcohol‐related problems (calculated from items 4 to 10; ordinal Cronbach's *⍺* = .87). Due to variation in the exact age when AUDIT was administered (*M* = 22.85 years, *SD* = 0.88, range = 21.16–26.48), age was regressed out of all three AUDIT scores (i.e. AUDIT‐Total, AUDIT‐Consumption, AUDIT‐Problem) before analyses were conducted to remove the possible effect of age on alcohol use.

#### Covariates

Covariates included family socioeconomic status at the first contact, ethnic origin of the twin pairs, and a set of variables measured at age 22 (relationship status, having child or not, living with parents or not, educational attainment, employment status). Sex was also included as a covariate when sex differences were not tested. Detailed information about measures of covariates is in Appendix S1. These covariates were selected because they have been associated with alcohol use in adulthood (e.g. Galvan & Caetano, 2003; Humensky, 2010; Leonard & Rothbard, 1999; Levy, Le Strat, Hoertel, Ancelet, & Dubertret, 2018).

### Analyses

All analytic code is available at https://github.com/tongc797/TEDS_Alcohol‐use. All analyses were preregistered on the Open Science Framework prior to accessing the data (https://osf.io/fjt3a). Deviations from the preregistered analysis plan are detailed and justified in Appendix S2. Analytic procedures for fitting the piecewise latent growth curve (LGC) model describing developmental trajectories of emotional and conduct problems are provided in Appendix S3.

#### Predicting alcohol use behaviors (phenotypic analyses)

Lavaan package 0.6‐12 (Rosseel, 2012) in R (R Core Team, 2022) was used for analyses. Cluster‐robust standard errors were used to account for the clustering of data within twin pairs. Full information maximum likelihood handled missing data based on missingness being predicted by sex, ethnicity, and family socioeconomic status, which is consistent with missingness at random (White, Royston, & Wood, 2011). Additionally, missingness of alcohol use variables were predicted by emotional and conduct problems (Table S1).

**Figure 1:**
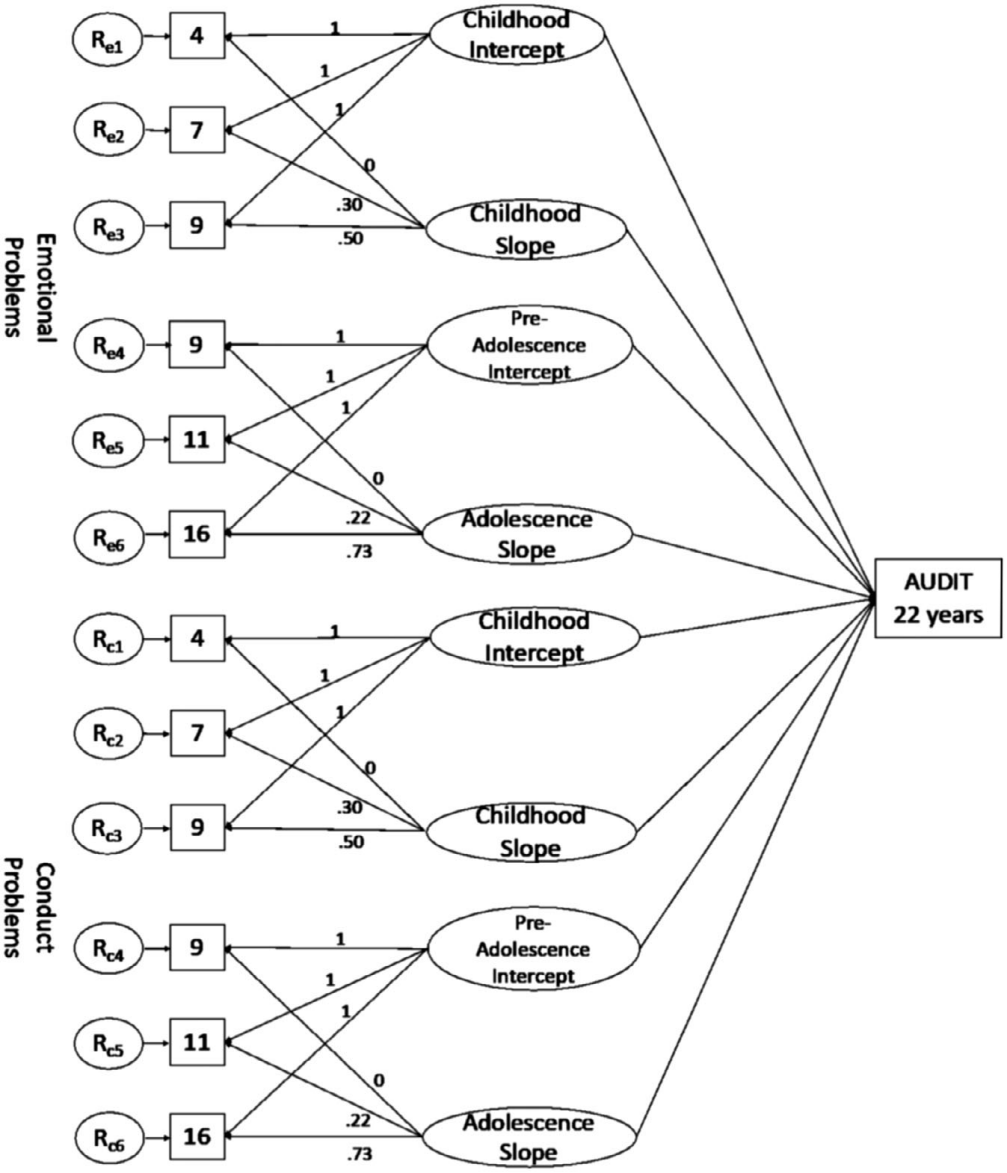
Piecewise latent growth factors of emotional and conduct problems predicting AUDIT (Alcohol Use Disorders Identification Test) scores at age 22 years (i.e. AUDIT‐Total, AUDIT‐Consumption, AUDIT‐Problem). Rectangles are measured emotional/conduct problems at ages 4, 7, 9, 11, and 16 years. Childhood symptoms (ages 4 to 9) are parent reports; adolescence symptoms (ages 9 to 16) are self‐reports. Factor loadings of the slope factors are determined by average ages reported at each wave. *R*_ei_ are residual variances for emotional problems, *R*_ci_ are residual variances for conduct problems. Covariances between all intercepts and slopes and between the residuals of emotional and conduct problems measured at the same age were estimated. Residuals between parent‐ and self‐reports at age 9 were also correlated. Non‐significant covariances were fixed to zero (if not significantly decreasing model fit)

Childhood intercept, childhood linear slope, preadolescence intercept, and adolescence linear slope of emotional and conduct problems derived from the piecewise LGC model simultaneously predicted AUDIT‐Total (Figure 1), along with the covariates. Next, sex differences in associations between emotional/conduct problems and alcohol use were tested by multi‐group analyses. We first constrained everything related to covariates to be equal across groups (i.e. female and male), except the regression coefficients of AUDIT‐Total on covariates. If the model fit was good, we then constrained parameters to be equal across groups in the following order (Wang et al., 2019): means of latent growth factors, variances of latent growth factors, covariances among latent growth factors, and residual variances and covariances of observed indicators. Chi‐square difference tests were used to investigate whether these constraints resulted in a significantly worse model fit. Parameters with the highest modification index were freed up one at a time, until the chi‐square difference test was nonsignificant. Finally, Wald tests were conducted to test whether regression coefficients of AUDIT‐Total on emotional/conduct problems and covariates were equivalent in males and females. After equating all possible regression coefficients, we tested whether the intercept and residual variance of AUDIT‐Total could be equated across groups.

As post hoc analyses, AUDIT‐Total was broken down into AUDIT‐Consumption and AUDIT‐Problem to understand whether results differed for *alcohol consumption* versus *alcohol‐related problems*. The same analytic procedures described above were conducted to estimate effects of emotional and conduct problems on AUDIT‐Consumption and AUDIT‐Problem. When conducting sex difference tests, parameters involving the latent growth factors were freed up across groups based on the final model we obtained for AUDIT‐Total, unless chi‐square difference tests suggested additional parameters needed to be freed. This was done to ensure the sex difference results were comparable for the three AUDIT scores, because changing the outcome should not influence the structure of the latent growth factors.

#### Predicting alcohol use behaviors (twin analyses)

The main aim of twin analyses was to test whether observed associations between emotional problems and alcohol use behaviors were best explained by emotional problems influencing alcohol use behaviors (i.e. direct paths), or by correlated genetic and environmental influences between emotional problems and alcohol use. Effects from conduct problems to alcohol use behaviors were controlled for. In this step of analyses, only significant predictors of alcohol use behaviors from the phenotypic analyses were retained, because decomposition of non‐significant covariances would inevitably be underpowered, is typically unstable, and is unlikely to yield interpretable results. Since we aimed to test for sex differences in both phenotypic and twin analyses, the presence of a significant effect in either sex in the phenotypic models was sufficient for a predictor to be included in the twin modeling part. Factor scores of the significant predictors (i.e. latent growth factors) were saved, and sex was regressed out of all variables. All analytic procedures were repeated for the three AUDIT scores (AUDIT‐Total, AUDIT‐Consumption, AUDIT‐Problem). All twin analyses were conducted via the OpenMx package 2.20.6 in R (Neale et al., 2016).

The classical twin design takes advantage of the fact that monozygotic (MZ) twins share 100% of their segregating genes and dizygotic (DZ) twins share on average 50%. Therefore, the difference in MZ and DZ twin pair correlations for behaviors can be used to infer genetic influences. Twins reared together in the same home share 100% of shared environmental influences (i.e. nongenetic influences that account for similarity among family members, including twin pairs). Nonshared environmental influences, defined as nongenetic influences that account for differences among family members, is the only component that contributes to differences in MZ twins. Based on these principles, twin studies can disentangle observed variances and covariances between multiple behaviors into genetic (A), shared (C), and nonshared environmental influences (E) (Rijsdijk & Sham, 2002).

**Figure 2:**
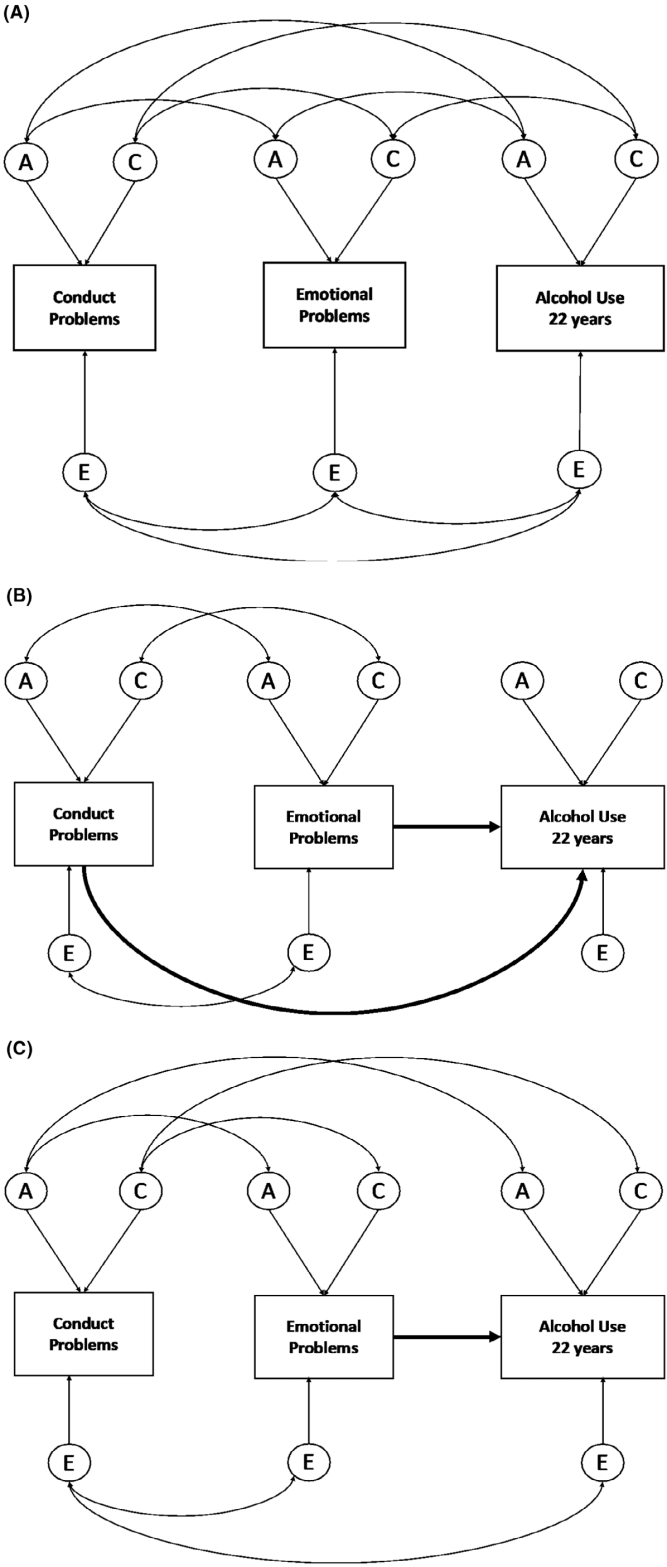
Twin model comparisons. Figure A represents the correlated factor solution of the Cholesky decomposition model with correlated A (additive genetic), C (shared environmental), and E (nonshared environmental) influences between the predictors (emotional and conduct problems) and alcohol use behaviors. Figure B represents the model with direct paths between the predictors and alcohol use behaviors. Figure C represents a hybrid model where conduct problems has correlated A, C, and E influences with alcohol use behaviors, while emotional problems has a direct path to alcohol use behaviors. In all models, the A, C, and E influences are correlated among the predictors (emotional and conduct problems)

We used twin data to compare several models (see examples demonstrated in Figure 2). The first model (Figure 2A) was a correlated factor solution of a Cholesky decomposition in which correlated A, C, and E influences between the predictors and alcohol use behaviors were specified, so did A, C, and E correlations among the predictors (Neale & Cardon, 1992). The second model (Figure 2B) incorporated direct paths between all the predictors and alcohol use behaviors (with correlated A, C, and E influences among the predictors). This model implies that the genetic and environmental influences on the predictors were transmitted to alcohol use behaviors via the direct paths (see Torvik et al., 2019; Turkheimer & Harden, 2014 for more details). If the model with direct paths (Figure 2B) had a lower Akaike Information Criterion (AIC) than the model with correlated A, C, and E factors (Figure 2A), we accepted the model with direct paths. This result would be consistent with the hypothesis that the predictors directly influenced alcohol use behaviors, rather than the predictors and alcohol use behaviors had correlated genetic and environmental influences. Alternatively, if the model with direct paths for all predictors (Figure 2B) had higher AIC than the model with correlated A, C, and E factors (Figure 2A), we tested which individual direct paths should be replaced by correlated A, C, and E influences to reach the best‐fitting model. For example, the effect of emotional problems on alcohol use may be best explained by a direct path, but the effect of conduct problems on alcohol use may be best explained by correlated A, C, and E factors (Figure 2C). Using the final best‐fitting model obtained, we examined sex differences in direct paths by testing whether they could be constrained to be equal in males and females.

## Results

### Descriptive statistics

Descriptive statistics for all study variables separated by sex are in Table 1. At all ages, females showed more emotional problems and males showed more conduct problems (*p*s < .001). At age 22, the mean AUDIT‐Total score was 8.90 for males (*SD* = 5.30) and 7.73 for females (*SD* = 4.82) (*p* < .001). Using the recommended cut‐off of eight for hazardous or harmful alcohol consumption (Saunders et al., 1993), 50% of the present sample was at risk for hazardous or harmful alcohol use at age 22. Correlations among study variables separated by sex are in Table S2.

**Table 1:** Descriptive statistics

	*n*	*M*	*SD*	Range	
				Min	Max
Male					
Emotional problems					
4 years (parent‐report)	7,460	2.00	1.74	0	10.00
7 years (parent‐report)	7,250	2.07	1.81	0	10.00
9 years (parent‐report)	3,146	1.55	1.83	0	10.00
9 years (self‐report)	3,084	3.02	2.30	0	10.00
11 years (self‐report)	5,382	1.97	1.96	0	10.00
16 years (self‐report)	4,397	1.94	1.86	0	10.00
Conduct problems					
4 years (parent‐ report)	7,464	2.26	1.61	0	10.00
7 years (parent‐report)	7,253	1.91	1.74	0	10.00
9 years (parent‐report)	3,147	1.47	1.56	0	10.00
9 years (self‐report)	3,083	2.45	1.94	0	10.00
11 years (self‐report)	5,383	2.15	1.77	0	10.00
16 years (self‐report)	4,396	1.74	1.50	0	9.00
AUDIT‐Total (22 years)	2,805	8.90	5.30	0	36.00
AUDIT‐Consumption (22 years)	2,805	6.37	2.71	0	12.00
AUDIT‐Problem (22 years)	2,805	2.16	2.80	0	20.57
Female					
Emotional problems					
4 years (parent‐report)	7,866	2.15	1.76	0	10.00
7 years (parent‐report)	7,695	2.33	1.87	0	10.00
9 years (parent‐report)	3,496	1.89	1.96	0	10.00
9 years (self‐report)	3,482	3.43	2.43	0	10.00
11 years (self‐report)	6,007	2.39	2.12	0	10.00
16 years (self‐report)	5,497	3.40	2.31	0	10.00
Conduct problems					
4 years (parent‐report)	7,867	1.92	1.51	0	10.00
7 years (parent‐report)	7,700	1.52	1.53	0	10.00
9 years (parent‐report)	3,495	1.13	1.32	0	10.00
9 years (self‐report)	3,485	2.00	1.75	0	10.00
11 years (self‐report)	6,008	1.67	1.52	0	10.00
16 years (self‐report)	5,496	1.58	1.44	0	10.00
AUDIT‐Total (22 years)	4,764	7.73	4.82	0	37.00
AUDIT‐Consumption (22 years)	4,766	5.65	2.56	0	12.00
AUDIT‐Problem (22 years)	4,763	1.79	2.52	0	20.57

Emotional and conduct problems ranged from 0 to 10 at all ages. AUDIT = Alcohol Use Disorders Identification Test. AUDIT‐Total score ranged from 0 to 40, AUDIT‐Consumption score ranged from 0 to 12, AUDIT‐Problem score ranged from 0 to 28.

### Predicting alcohol use behaviors

Results for the piecewise LGC model without alcohol use behaviors as outcomes are provided in Appendix S3.

#### Overall alcohol use (AUDIT‐Total)

**Figure 3:**
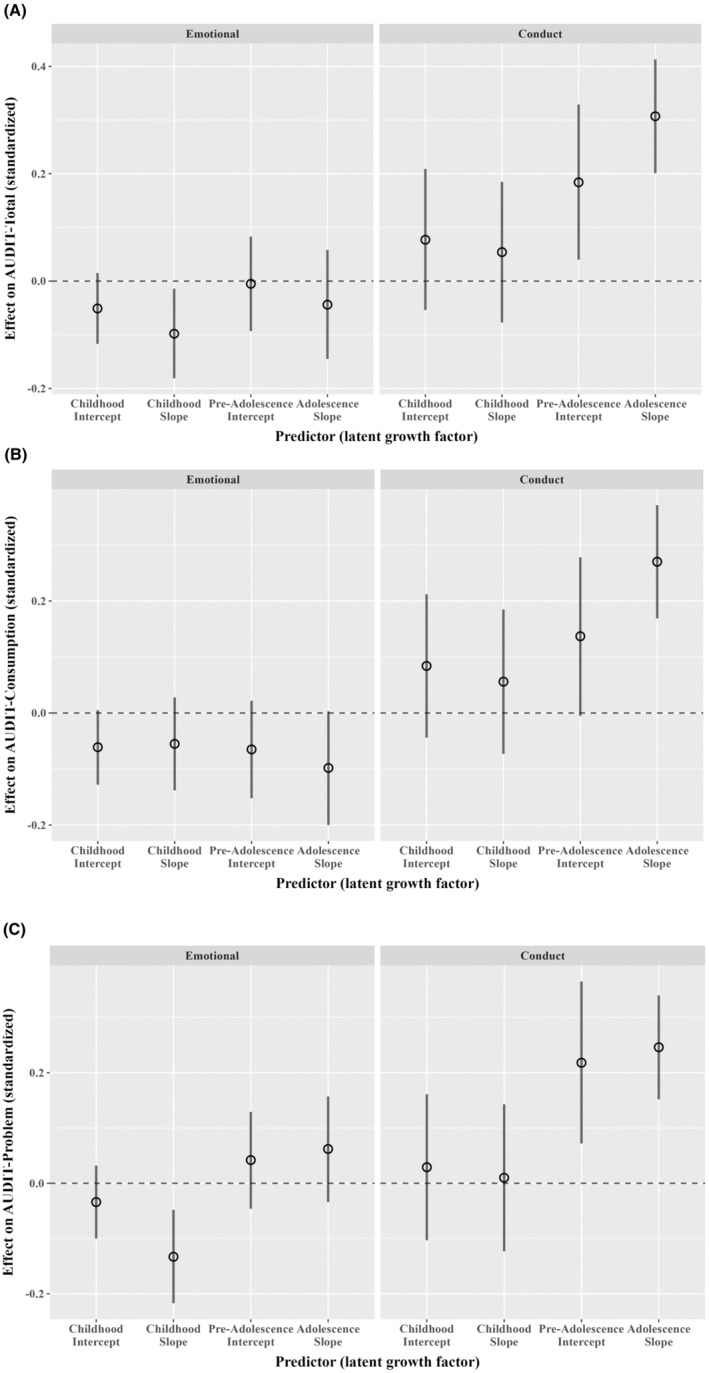
Latent growth factors of emotional and conduct problems predicting Alcohol Use Disorders Identification Test (AUDIT) scores (phenotypic model). Standardized path coefficients and 95% confidence intervals are displayed

Figure 3A shows estimates of the regression paths from the intercepts and linear slope factors of emotional and conduct problems to AUDIT‐Total. The childhood linear slope of emotional problems was negatively associated with AUDIT‐Total (*β* = −.10, *p* = .02). The preadolescence intercept and the adolescence linear slope of conduct problems were positively associated with AUDIT‐Total (intercept: *β* = .18, *p* = .01; slope: *β* = .31, *p* < .001). Testing sex differences indicated that the regression coefficient of the preadolescence intercept of emotional problems was significantly higher (*p* = .01) in females (*β* = .03, *p* = .48) than in males (*β* = −.07, *p* = .16), although neither was significantly different from zero. Therefore, we concluded that no meaningful sex differences were found. Regression coefficients estimated from the multi‐group model with males and females are in Figure S1a. Model fit indices are presented in Table S3.

**Figure 4:**
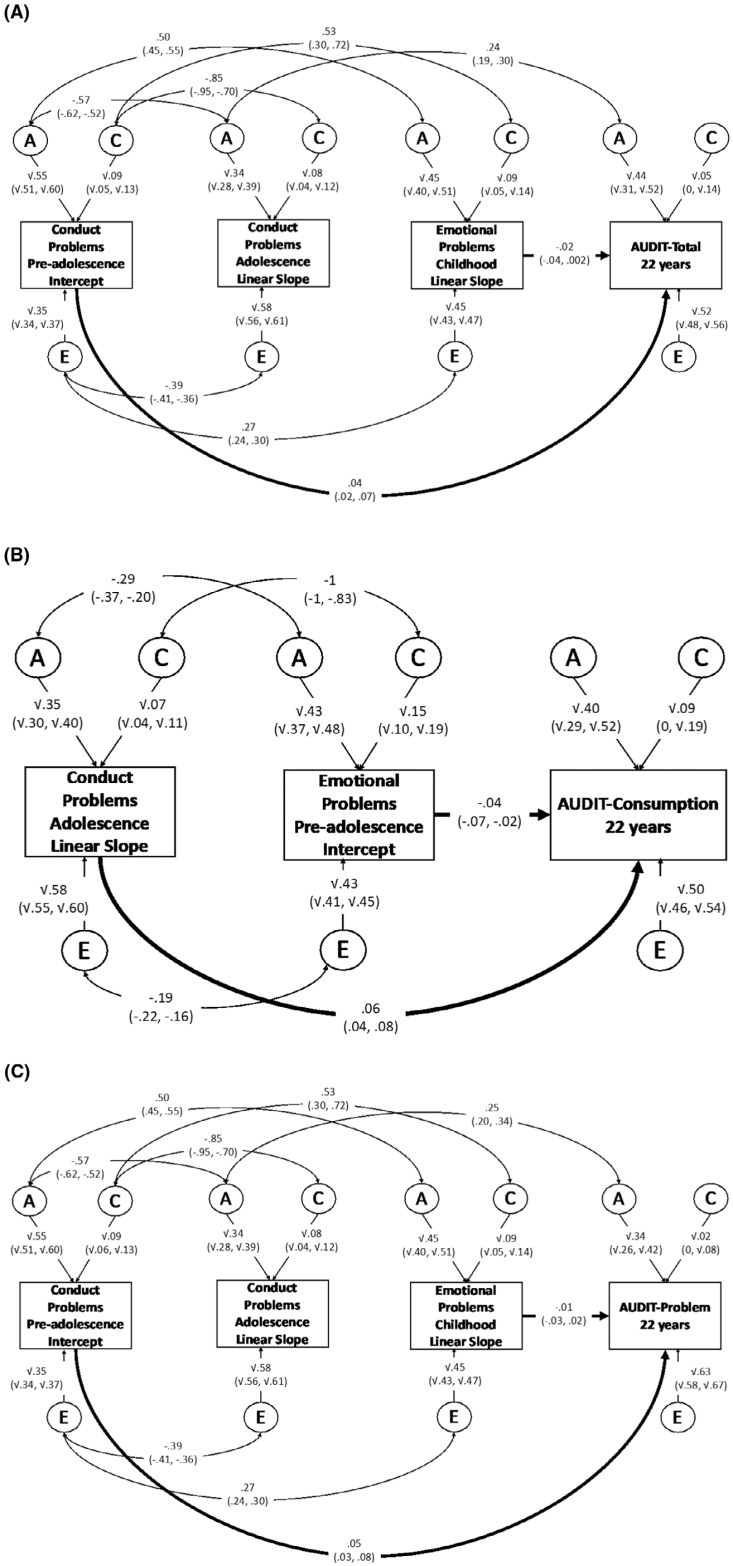
Final twin models obtained for three scores from the Alcohol Use Disorders Identification Test (AUDIT‐Total, AUDIT‐Consumption, AUDIT‐Problem). A = additive genetic influences, C = shared environmental influences, E = nonshared environmental influences. In all models, non‐significant correlations among the predictors are dropped based on results from the piecewise latent growth curve model. Bold single‐headed arrows show standardized estimates of direct paths, plain single‐headed arrows show A, C, E contributions to each variable. Double‐headed arrows show the correlations of A, C, and E factors among the variables. 95% CIs are in parentheses

Using twin models, we tested whether the significant association between the childhood linear slope of emotional problems and AUDIT‐Total was better explained by a direct path or by correlated genetic and environmental influences. Effects of the preadolescence intercept of conduct problems and the adolescence linear slope of conduct problems on AUDIT‐Total were controlled since they were both significant. Figure 4A shows the final model that had lower AIC value than the model with correlated A, C, and E influences among all predictors and AUDIT‐Total (ΔAIC = −1.29). In this final model, although the effect of the childhood linear slope of emotional problems on AUDIT‐Total was best explained by a direct path, the effect itself was non‐significant. Notably, in this final model, the association between the adolescence linear slope of conduct problems and AUDIT‐Total was captured via a correlated genetic path rather than a direct path, as suggested by AIC values for model fit. By comparing a series of model results (see details in Figure S2), we determined that changing the direct path from the adolescence linear slope of conduct problems to AUDIT‐Total to a correlated genetic path made the effect of childhood linear slope of emotional problems on AUDIT‐Total become non‐significant, suggesting that assumptions made on covariates (i.e. conduct problems) have influences on estimates related to our predictor of interest (i.e. emotional problems). In other words, the implicit assumption in the phenotypic model that all effects from emotional and conduct problems were direct phenotypic influences did not hold for the adolescence linear slope of conduct problems, of which the association was better explained by correlated genetic influences with AUDIT‐Total. In summary, the twin modeling results suggested that the significant effect of childhood emotional problems on AUDIT‐Total in the phenotypic model may be spurious, resulting from inaccurately accounting for ‘direct’ effects from conduct problems that may not exist. Finally, no sex differences were found for the direct paths from any predictor to AUDIT‐Total. Details about model fit indices and estimates of all the models tested are in Table S4 and Figure S2.

#### Alcohol consumption (AUDIT‐consumption)

To further explore whether effects of emotional and conduct problems differed for alcohol consumption versus alcohol‐related problems, we repeated the analyses with AUDIT‐Consumption and AUDIT‐Problem as outcomes. Figure 3B shows estimates of the regression paths from the intercepts and linear slope factors of emotional and conduct problems to AUDIT‐Consumption. Only the adolescence linear slope of conduct problems was positively associated with AUDIT‐Consumption (*β* = .27, *p* < .001). Tests of sex differences suggested that the preadolescence intercept of emotional problems was negatively associated with AUDIT‐Consumption in males (*β* = −.13, *p* = .01) but not in females (*β* = −.02, *p* = .64). A Wald test suggested that the regression coefficients for males and females were significantly different (*p* = .004). Regression coefficients estimated from the multi‐group model with males and females are in Figure S1b. Model fit indices are presented in Table S3.

We retained the preadolescence intercept of emotional problems in twin analyses, controlling for the effect of the adolescence linear slope of conduct problems on AUDIT‐Consumption. The final model we obtained (Figure 4B) suggested that the association between the preadolescence intercept of emotional problems and AUDIT‐Consumption was best explained by direct paths. The model had a lower AIC value than the model with correlated A, C, and E influences among all predictors and AUDIT‐Consumption (ΔAIC = −3.81). Tests for sex differences suggested that the direct path from the preadolescent intercept of emotional problems to AUDIT‐Consumption was only significant for males (*β* = −.08, 95% CI [−0.12, −0.04]) but not for females (*β* = −.02, 95% CI [−0.05, 0.01]), consistent with findings from the phenotypic models. Details about model fit indices and estimates of all the models tested are in Table S4 and Figure S3.

#### Alcohol‐related problems (AUDIT‐problem)

AUDIT‐Problem scores were log transformed because it was skewed. Figure 3C shows estimates of the regression paths from the intercepts and linear slope factors of emotional and conduct problems to AUDIT‐Problem. The effects of emotional and conduct problems on AUDIT‐Problem were very similar to the results obtained for AUDIT‐Total (emotional childhood slope: *β* = −.13, *p* = .002; conduct preadolescence intercept: *β* = .22, *p* = .004; conduct adolescence slope: *β* = .25, *p* < .001). No sex differences were found for AUDIT‐Problem (see estimates in Figure S1c). Model fit indices are presented in Table S3.

Results from twin analyses were also very similar to the results for AUDIT‐Total (Figure 4C). The final model we obtained had lower AIC value than the model with correlated A, C, and E influences among all predictors and AUDIT‐Problem (ΔAIC = −6.77). The effect of the childhood linear slope of emotional problems on AUDIT‐Problem was best explained by a direct path but the effect was non‐significant. This was likely due to the association between the adolescence linear slope of conduct problems and AUDIT‐Problem being explained by correlated genetic influences rather than a direct path. No sex differences were found for the direct paths from any predictor to AUDIT‐Problem. Details about model fit indices and estimates of all the models tested are in Table S4 and Figure S4.

## Discussion

In the present study, we found that when controlling for conduct problems, an increase in emotional problems during childhood was associated with less *alcohol‐related problems* in early adulthood, although this association became non‐significant when we only controlled for direct influences from conduct problems to alcohol‐related problems, excluding effects that were better explained by correlated genetic factors. Emotional problems in preadolescence were associated with less early adult *alcohol consumption* in males, when controlling for direct influences from conduct problems.

Our findings are consistent with previous literature, whereby emotional problems were associated with less alcohol use after controlling for externalizing problems (e.g. Foster et al., 2018; Maggs et al., 2008; Pitkanen, Kokko, Lyyra, & Pulkkinen, 2008). Such findings suggest that emotional problems were *uniquely* associated with *less* alcohol use above and beyond risk pathways to alcohol use operating through externalizing problems (Hussong et al., 2017). Twin analyses further suggested that such effects were unlikely explained by correlated genetic or environmental influences. Given that drinking alcohol is common in early adulthood and may serve as an important way of socializing with peers (Schulenberg & Maggs, 2001), interpersonal difficulties associated with emotional problems may prevent early adults from engaging in these social contexts (Nelson et al., 2008). Moreover, our findings suggest that such process may start during transition to adolescence. Children with high levels of emotional problems at the transition to adolescence may be less socially integrated or select peers who engage in less risky behaviors such as alcohol use, and then continue to hang out with peer groups that share the same tendencies across adolescence and early adulthood (Osgood, Feinberg, Wallace, & Moody, 2014; Schulenberg & Maggs, 2001). Finally, although we were mainly interested in the unique effect of emotional problems on alcohol use beyond risks associated with conduct problems, we acknowledge the possibility that in very specific cases, if emotional problems were associated with more conduct problems (see Figure S5), emotional problems might be indirectly associated with *more severe* alcohol use through conduct problems.

The sex difference we found for the negative association between emotional problems and *alcohol consumption* is intriguing and worth further investigation. Previous research suggested that due to gender role socialization, adolescent boys are more susceptible to influences from peers for risk‐taking behaviors than girls (McCoy, Dimler, Samuels, & Natsuaki, 2019). Therefore, high levels of emotional problems may counteract peer influences on risk‐taking behaviors (e.g. drinking) in males during adolescence and early adulthood.

It is surprising that the significant association between the slope of childhood emotional problems and early adult *alcohol‐related problems* did not hold in the final model in our twin analyses. This was not due to this association being explained by genetic or environmental confounds, but due to the discovery that some effects of conduct problems on alcohol‐related problems were confounded by shared genetic influences, and therefore, should not be controlled. We demonstrated in our series of twin models that changing the assumption on effects of conduct problems also changed the effects of emotional problems. Our findings suggest that when examining the unique association between emotional problems and alcohol use behaviors, it is important to control for externalizing problems, but it is equally important to correctly control for effects that reflect direct phenotypic influences while accounting for effects that reflect correlated genetic or environmental influences. Inaccurately controlling for ‘direct’ effects that may not exist can lead to spurious findings.

### Limitations and conclusions

Our findings should be interpreted with several limitations. First, attrition analysis suggested differences in sample characteristics between participants with data at age 22 versus those without, suggesting that our sample may be biased toward the population with fewer behavioral problems or less alcohol use; however, including variables associated with missingness helped mitigate biases in parameter estimates (Nicholson, Deboeck, & Howard, 2017). Second, emotional and conduct problems were not measured between 11 and 16 years, thus developmental changes during this important window could be missed. Third, we used parent reports during childhood, and self‐reports during adolescence. Although this shift is developmentally appropriate, the developmental timing effects could be confounded by rater differences. Measures at some ages (e.g. 9 years) also had lower reliability than others. Additionally, self‐reported symptoms were higher than parent reports, although this should not affect the individual differences we examined. Fourth, the use of modification indices in searching for the best‐fitting multi‐group models when testing sex differences would inevitably lead to over‐fitting.

In conclusion, when controlling for conduct problems, children who increased faster in emotional problems across childhood reported having less *alcohol‐related problems* in early adulthood. However, this effect of emotional problems disappeared when only direct influences from conduct problems to alcohol‐related problems were controlled. When controlling for direct influences of conduct problems, males with higher levels of emotional problems at age 9 reported less *alcohol consumption* in early adulthood, and this association was unlikely confounded by correlated genetic or environmental influences. Overall, findings suggested that emotional problems in childhood and adolescence may not be important risk factors for early adult alcohol use above and beyond risk pathways operating through conduct problems; associations between emotional problems and later alcohol use are dependent on developmental timing, sex, and specific alcohol use outcomes.

## Ethical considerations

Ethical approval was received from the King's College London Research Ethics Committee (reference: PNM/09/10‐104). Informed consent was obtained from all participating parents and adult twins. The present study was deemed as non‐human subject research by the institutional review board at The Pennsylvania State University.

**Key points**

- Prior research is unclear whether emotional problems during childhood and adolescence are longitudinally associated with early adult alcohol use behaviors.
- Co‐occurring conduct problems, developmental change/timing of emotional problems, and sex differences may explain mixed findings. Associations between emotional problems and alcohol use behaviors may be explained by correlated genetic/environmental influences.
- When accounting for direct influences from conduct problems, emotional problems were not associated with alcohol‐related problems during early adulthood, but emotional problems at age 9 were negatively associated with alcohol consumption in males.
- Emotional problems in childhood and adolescence were associated with less early adult alcohol use or not associated at all, depending on sex, developmental timing, and specific alcohol use outcomes. Future research should examine mechanisms explaining such findings.

## Supporting information


**Table S1.** Comparison of participants with versus without data at age 22 (total *N* = 19,908).
**Table S2.** Correlations among all study variables by sex.
**Table S3.** Fit indices for models predicting AUDIT scores.
**Table S4.** Model comparison results for different twin models.
**Table S5.** Estimates of variances (unstandardized) and covariances (standardized) from the full piecewise latent growth curve model.
**Figure S1.** Multi‐group analyses by sex results for latent growth factors of emotional and conduct problems predicting alcohol use disorders identification test (AUDIT) scores (phenotypic model).
**Figure S2.** Twin model results for AUDIT‐Total (Alcohol Use Disorders Identification Test – Total score).
**Figure S3.** Twin model results for AUDIT‐Consumption (Alcohol Use Disorders Identification Test – Consumption).
**Figure S4.** Twin model results for AUDIT‐Problem (Alcohol Use Disorders Identification Test – Problem).
**Figure S5.** Piecewise latent growth curve model results (phenotypic model).
**Figure S6.** The mean trends of emotional and conduct problems across ages 4 to 16 years for males (a) and females (b).
**Appendix S1.** Covariate measures.
**Appendix S2.** Deviations from preregistration.
**Appendix S3.** Methods and results for piecewise latent growth curve (LGC) models.

## Data Availability

Data used for this submission may be made available on request to the Twins Early Development Study (TEDS), through their data access mechanism (see www.teds.ac.uk/researchers/teds‐data‐access‐policy).
