# Supplementary material for: Developmental trajectories of child and adolescent emotional problems: associations with early adult alcohol use behaviors
Source: J Child Psychol Psychiatry. 2024 Jun 26;66(1):85–97. doi: 10.1111/jcpp.14034 (PMC11652264; doi:10.1111/jcpp.14034)
Supplement: Supplementary file 1 — Table S1. Comparison of participants with versus without data at age 22 (total N = 19,908). Table S2. Correlations among all study variables by sex. Table S3. Fit indices for models predicting AUDIT scores. Table S4. Model comparison results for different twin models. Table S5. Estimates of variances (unstandardized) and covariances (standardized) from the full piecewise latent growth curve model. Figure S1. Multi‐group analyses by sex results for latent growth factors of emotional and conduct problems predicting alcohol use disorders identification test (AUDIT) scores (phenotypic model). Figure S2. Twin model results for AUDIT‐Total (Alcohol Use Disorders Identification Test – Total score). Figure S3. Twin model results for AUDIT‐Consumption (Alcohol Use Disorders Identification Test – Consumption). Figure S4. Twin model results for AUDIT‐Problem (Alcohol Use Disorders Identification Test – Problem). Figure S5. Piecewise latent growth curve model results (phenotypic model). Figure S6. The mean trends of emotional and conduct problems across ages 4 to 16 years for males (a) and females (b). Appendix S1. Covariate measures. Appendix S2. Deviations from preregistration. Appendix S3. Methods and results for piecewise latent growth curve (LGC) models. [file JCPP-66-85-s001.docx]

**Supporting Information**

**Table S1.** Comparison of participants with versus without data at age 22 (total *N* = 19908).

|  | **Participants with data at age 22 (*n* = 7569)** | **Participants without data at age 22 (*n* = 12339)** | **Difference test** |
| --- | --- | --- | --- |
| Female | 62.9% | 44.2% | *χ^2^*(1) = 657.75, *p* < .001 |
| White | 93.9% | 92.1% | *χ^2^*(1) = 22.49, *p* < .001 |
| Family socio-economic status at initial contact | *M* = .28 | *M* = -.05 | *t*(15,293) = 22.14, *p* < .001 |
| Emotional problems at age 4 (parent-report) | *M* = 2.05 | *M* = 2.09 | *t*(13,082) = -1.65, *p* = .10 |
| Emotional problems at age 7 (parent-report) | *M* = 2.14 | *M* = 2.24 | *t*(13,582) = -3.35, *p* < .001 |
| Emotional problems at age 9 (parent-report) | *M* = 1.67 | *M* = 1.78 | *t*(6,640) = -2.27, *p* = .02 |
| Emotional problems at age 9 (self-report) | *M* = 3.19 | *M* = 3.28 | *t*(6,564) = -1.60, *p* = .11 |
| Emotional problems at age 11 (self-report) | *M* = 2.18 | *M* = 2.19 | *t*(11,387) = -.23, *p* = .82 |
| Emotional problems at age 16 (self-report) | *M* = 2.83 | *M* = 2.64 | *t*(9,892) = 4.12, *p* < .001 |
| Conduct problems at age 4 (parent-report) | *M* = 1.89 | *M* = 2.21 | *t*(13,282) = -12.54, *p* < .001 |
| Conduct problems at age 7 (parent-report) | *M* = 1.47 | *M* = 1.87 | *t*(14,252) = -15.09, *p* < .001 |
| Conduct problems at age 9 (parent-report) | *M* = 1.13 | *M* = 1.42 | *t*(6,598) = -8.03, *p* < .001 |
| Conduct problems at age 9 (self-report) | *M* = 2.04 | *M* = 2.35 | *t*(6,487) = -6.82, *p* < .001 |
| Conduct problems at age 11 (self-report) | *M* = 1.68 | *M* = 2.11 | *t*(11,302) = -13.91, *p* < .001 |
| Conduct problems at age 16 (self-report) | *M* = 1.52 | *M* = 1.82 | *t*(8,606) = -9.85, *p* < .001 |

*Notes:* Family socio-economic status were standardized scores. Emotional and conduct problems ranged from 0-10 at all ages.

**Table S2.** Correlations among all study variables by sex.

| **Male** | | | | | | | | | | | | | |  | |  | |
| --- | --- | --- | --- | --- | --- | --- | --- | --- | --- | --- | --- | --- | --- | --- | --- | --- | --- |
|  | 1 | 2 | 3 | 4 | 5 | 6 | 7 | 8 | 9 | 10 | 11 | 12 | 13 | | 14 | | 15 |
| 1 Emo-4 (P) | 1 |  |  |  |  |  |  |  |  |  |  |  |  | |  | |  |
| 2 Emo-7 (P) | .40^**^ | 1 |  |  |  |  |  |  |  |  |  |  |  | |  | |  |
| 3 Emo-9 (P) | .36^**^ | .49^**^ | 1 |  |  |  |  |  |  |  |  |  |  | |  | |  |
| 4 Emo-9 (S) | .14^**^ | .20^**^ | .36^**^ | 1 |  |  |  |  |  |  |  |  |  | |  | |  |
| 5 Emo-11 (S) | .14^**^ | .22^**^ | .27^**^ | .38^**^ | 1 |  |  |  |  |  |  |  |  | |  | |  |
| 6 Emo-16 (S) | .12^**^ | .15^**^ | .18^**^ | .24^**^ | .32^**^ | 1 |  |  |  |  |  |  |  | |  | |  |
| 7 Con-4 (P) | .22^**^ | .17^**^ | .20^**^ | .15^**^ | .11^**^ | .06^**^ | 1 |  |  |  |  |  |  | |  | |  |
| 8 Con-7 (P) | .15^**^ | .26^**^ | .25^**^ | .16^**^ | .11^**^ | .05^**^ | .50^**^ | 1 |  |  |  |  |  | |  | |  |
| 9 Con-9 (P) | .17^**^ | .20^**^ | .35^**^ | .19^**^ | .13^**^ | .10^**^ | .41^**^ | .56^**^ | 1 |  |  |  |  | |  | |  |
| 10 Con-9 (S) | .05^*^ | .09^**^ | .16^**^ | .33^**^ | .16^**^ | .09^**^ | .25^**^ | .34^**^ | .42^**^ | 1 |  |  |  | |  | |  |
| 11 Con-11 (S) | .05^**^ | .09^**^ | .17^**^ | .21^**^ | .33^**^ | .11^**^ | .26^**^ | .34^**^ | .38^**^ | .40^**^ | 1 |  |  | |  | |  |
| 12 Con-16 (S) | .03 | .05^**^ | .12^**^ | .11^**^ | .13^**^ | .26^**^ | .17^**^ | .19^**^ | .22^**^ | .23^**^ | .35^**^ | 1 |  | |  | |  |
| 13 AUDIT-Total | -.03 | -.07^**^ | -.02 | -.06^*^ | -.07^**^ | .02 | .01 | -.01 | .04 | .04 | .03 | .14^**^ | 1 | |  | |  |
| 14 AUDIT-Consumption | -.05^*^ | -.10^**^ | -.05 | -.10^**^ | -.11^**^ | -.03 | -.01 | -.04^*^ | .01 | .02 | .01 | .10^**^ | .86^**^ | | 1 | |  |
| 15 AUDIT-Problem | -.01 | -.04 | .01 | -.01 | -.02 | .06^*^ | .01 | .02 | .06^*^ | .06 | .05^*^ | .14^**^ | .91^**^ | | .56^**^ | | 1 |
| **Female** |  |  |  |  |  |  |  |  |  |  |  |  |  | |  | |  |
| 1 Emo-4 (P) | 1 |  |  |  |  |  |  |  |  |  |  |  |  | |  | |  |
| 2 Emo-7 (P) | .40^**^ | 1 |  |  |  |  |  |  |  |  |  |  |  | |  | |  |
| 3 Emo-9 (P) | .37^**^ | .52^**^ | 1 |  |  |  |  |  |  |  |  |  |  | |  | |  |
| 4 Emo-9 (S) | .18^**^ | .28^**^ | .39^**^ | 1 |  |  |  |  |  |  |  |  |  | |  | |  |
| 5 Emo-11 (S) | .16^**^ | .25^**^ | .28^**^ | .41^**^ | 1 |  |  |  |  |  |  |  |  | |  | |  |
| 6 Emo-16 (S) | .08^**^ | .18^**^ | .21^**^ | .24^**^ | .36^**^ | 1 |  |  |  |  |  |  |  | |  | |  |
| 7 Con-4 (P) | .24^**^ | .19^**^ | .22^**^ | .19^**^ | .13^**^ | .07^**^ | 1 |  |  |  |  |  |  | |  | |  |
| 8 Con-7 (P) | .16^**^ | .26^**^ | .22^**^ | .19^**^ | .18^**^ | .08^**^ | .46^**^ | 1 |  |  |  |  |  | |  | |  |
| 9 Con-9 (P) | .14^**^ | .17^**^ | .30^**^ | .23^**^ | .19^**^ | .12^**^ | .41^**^ | .53^**^ | 1 |  |  |  |  | |  | |  |
| 10 Con-9 (S) | .07^**^ | .08^**^ | .15^**^ | .37^**^ | .23^**^ | .10^**^ | .24^**^ | .34^**^ | .43^**^ | 1 |  |  |  | |  | |  |
| 11 Con-11 (S) | .07^**^ | .10^**^ | .14^**^ | .26^**^ | .37^**^ | .14^**^ | .24^**^ | .31^**^ | .36^**^ | .40^**^ | 1 |  |  | |  | |  |
| 12 Con-16 (S) | .01 | .08^**^ | .13^**^ | .13^**^ | .15^**^ | .25^**^ | .19^**^ | .21^**^ | .26^**^ | .23^**^ | .34^**^ | 1 |  | |  | |  |
| 13 AUDIT-Total | -.03^*^ | -.03 | -.03 | .02 | -.01 | .03 | .02 | 0 | .05^*^ | .03 | .04^*^ | .15^**^ | 1 | |  | |  |
| 14 AUDIT-Consumption | -.06^**^ | -.04^*^ | -.05^*^ | 0 | -.03 | -.01 | 0 | 0 | .03 | 0 | .02 | .11^**^ | .86^**^ | | 1 | |  |
| 15 AUDIT-Problem | -.01 | -.01 | -.01 | .03 | .02 | .06^**^ | .02 | 0 | .06^*^ | .04 | .05^**^ | .15^**^ | .89^**^ | | .53^**^ | | 1 |

*Notes:* Emo = emotional problems, Con = conduct problems, P = parent-report, S = self-report. Numbers in variable names represent the age (in years) when it was measured. AUDIT = Alcohol Use Disorders Identification Test. ^**^*p* < .01, ^*^*p* < .05.

**Table S3.** Fit indices for models predicting AUDIT scores.

| Model | *𝜒 ^2^* | df | *p* | RMSEA | CFI | TLI | SRMR |
| --- | --- | --- | --- | --- | --- | --- | --- |
| **Full sample** |  |  |  |  |  |  |  |
| AUDIT-Total as outcome | 159.65 | 70 | <.001 | .01 | 1.00 | .99 | .01 |
| AUDIT-Consumption as outcome | 159.89 | 70 | <.001 | .01 | 1.00 | .99 | .01 |
| AUDIT-Problem as outcome | 158.35 | 70 | <.001 | .01 | 1.00 | .99 | .01 |
| **Multi-group analyses by sex** | | | | | | | |
| AUDIT-Total as outcome | 811.76 | 292 | <.001 | .01 | .98 | .97 | .03 |
| AUDIT-Consumption as outcome | 815.30 | 292 | <.001 | .01 | .98 | .97 | .03 |
| AUDIT-Problem as outcome | 816.77 | 294 | <.001 | .01 | .98 | .97 | .03 |

*Notes:* *𝜒 ^2^* represents robust model test statistics. RMSEA = root mean square error of approximation, CFI = comparative fit index, TLI = Tucker-Lewis index, SRMR = standardized root mean residual. AUDIT = Alcohol Use Disorders Identification Test.

**Table S4.** Model comparison results for different twin models.

| **Outcome: AUDIT-Total** | -2LL | *df* | AIC |
| --- | --- | --- | --- |
| Correlated A, C, E factors (Figure S2a) | 157495.38 | 66265 | 157557.38 |
| All predictors have direct paths (Figure S2b) | 157528.86 | 66271 | 157578.86 |
| Conduct problems adolescence linear slope has correlated paths, other predictors have direct paths (Figure S2c) | 157502.90 | 66269 | 157556.90 |
| Fix C and E covariance between conduct problems adolescence linear slope and AUDIT-Total to 0 based on Figure S2c model (Figure S2d) | 157506.09 | 66271 | 157556.09 |
| **Outcome: AUDIT-Consumption** | -2LL | *df* | AIC |
| Correlated A, C, E factors (Figure S3a) | 127269.65 | 46701 | 127311.65 |
| All predictors have direct paths (Figure S3b) | 127273.84 | 46705 | 127307.84 |
| **Outcome: AUDIT-Problem** | -2LL | *df* | AIC |
| Correlated A, C, E factors (Figure S4a) | 159225.02 | 66264 | 159287.02 |
| All predictors have direct paths (Figure S4b) | 159256.94 | 66270 | 159306.94 |
| Conduct problems adolescence linear slope has correlated paths, other predictors have direct paths (Figure S4c) | 159229.54 | 66268 | 159283.54 |
| Fix C and E covariance between conduct problems adolescent linear slope and AUDIT-Problem to 0 based on Figure S4c model (Figure S4d) | 159230.25 | 66270 | 159280.25 |

*Notes:* AUDIT = Alcohol Use Disorders Identification Test. A = additive genetic, C = shared environmental, E = nonshared environmental. LL = log likelihood, AIC = Akaike Information Criterion.

**Table S5.** Estimates of variances (unstandardized) and covariances (standardized) from the full piecewise latent growth curve model.

|  | 1 | 2 | 3 | 4 | 5 | 6 | 7 | 8 |
| --- | --- | --- | --- | --- | --- | --- | --- | --- |
| 1 Int-Chd-E | 1.41^**^  (1.23, 1.60) |  |  |  |  |  |  |  |
| 2 Slo-Chd-E | -.11  (-.28, .06) | 4.46^**^  (3.02, 5.90) |  |  |  |  |  |  |
| 3 Int-Ado-E | .38^**^  (.33, .44) | .39^**^  (.30, .48) | 2.34^**^  (2.15, 2.52) |  |  |  |  |  |
| 4 Slo-Ado-E | -.14^**^  (-.21, -.07) | -.08  (-.19, .03) | -.46^**^  (-.51, -.40) | 4.38^**^  (3.01, 5.74) |  |  |  |  |
| 5 Int-Chd-C | .30^**^  (.24, .37) | .11  (-.01, .22) | .28^**^  (.24, .32) | -.22^**^  (-.29, -.15) | 1.66^**^  (1.51, 1.80) |  |  |  |
| 6 Slo-Chd-C | -.05  (-.16, .06) | .11  (-.06, .28) | .05  (-.01, .12) | -.02  (-.11, .07) | -.44^**^  (-.51, -.36) | 4.62^**^  (3.64, 5.60) |  |  |
| 7 Int-Ado-C | .13^**^  (.09, .18) | .16^**^  (.08, .23) | .51^**^  (.45, .56) | -.42^**^  (-.50, -.33) | .52^**^  (.48, .56) | .29^**^  (.22, .37) | 1.63^**^  (1.50, 1.76) |  |
| 8 Slo-Ado-C | -.09^**^  (-.14, -.03) | .05  (-.04, .14) | -.29^**^  (-.36, -.22) | .46^**^  (.38, .63) | -.23^**^  (-.29, -.17) | -.21^**^  (-.29, -.12) | -.57^**^  (-.61, -.52) | 3.08^**^  (2.32, 3.85) |

*Notes:* Model fit indices: 𝜒^2^ (20) = 39.22; *p* = .01; RMSEA = .01; CFI = 1.00; TLI = 1.00; SRMR = .01. ^**^*p* < .01; ^*^*p* < .05. 95% confidence intervals are in parentheses. Int = intercept, Slo = slope, Chd = childhood, Ado = adolescence, E = emotional problems, C = conduct problems.

**(a)**

**
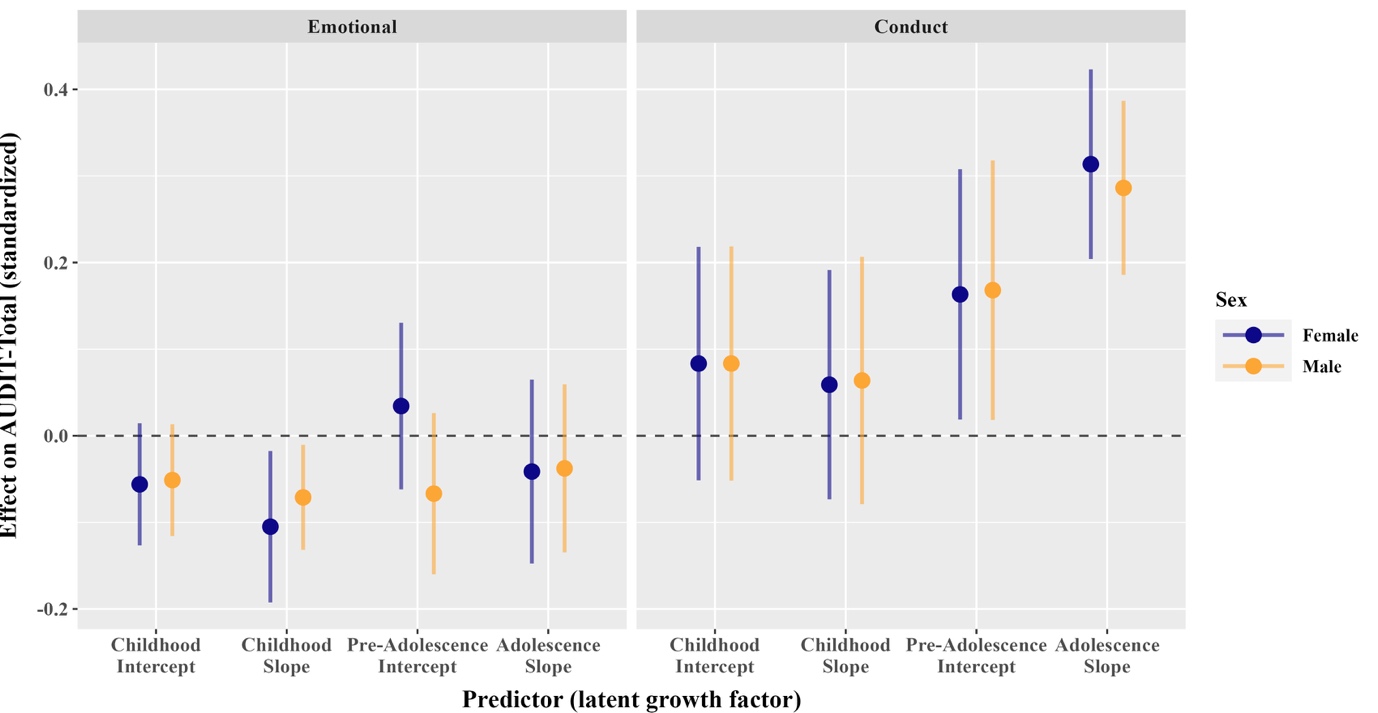
**

**(b)**

**
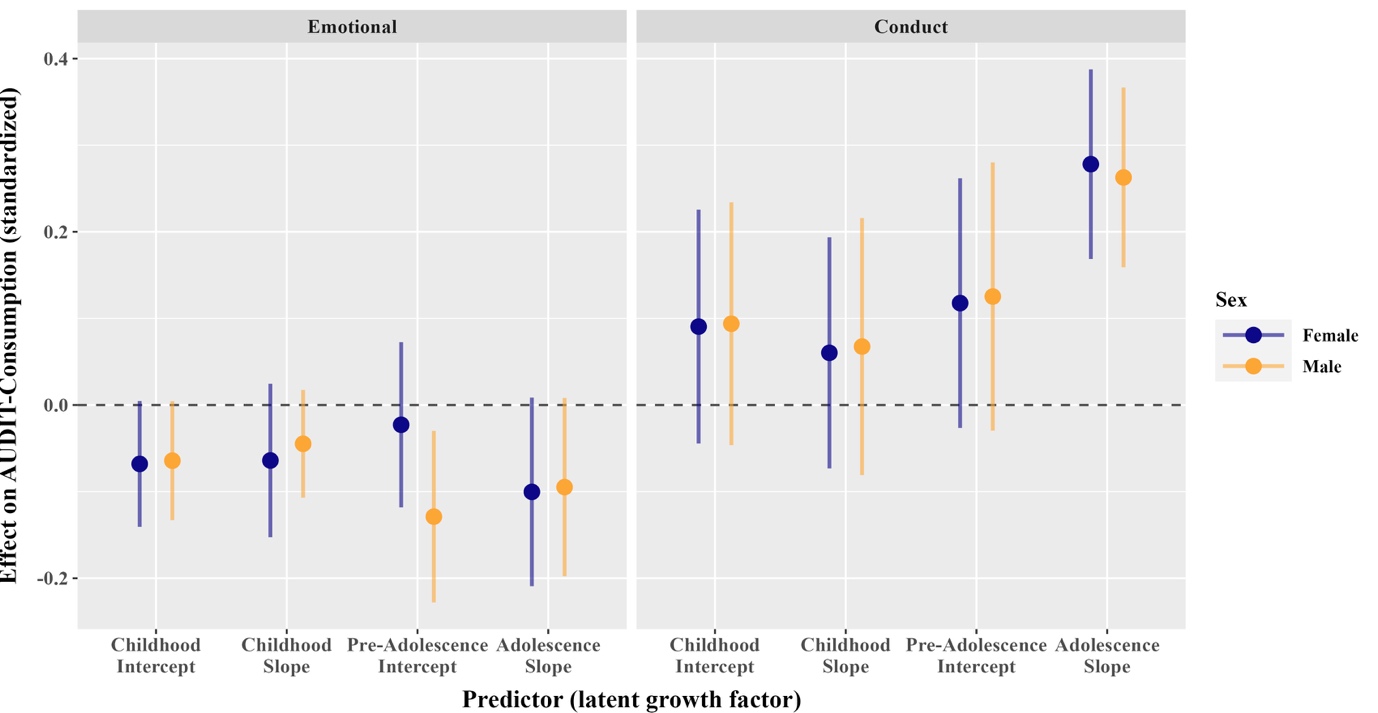
**

**(c)**

**
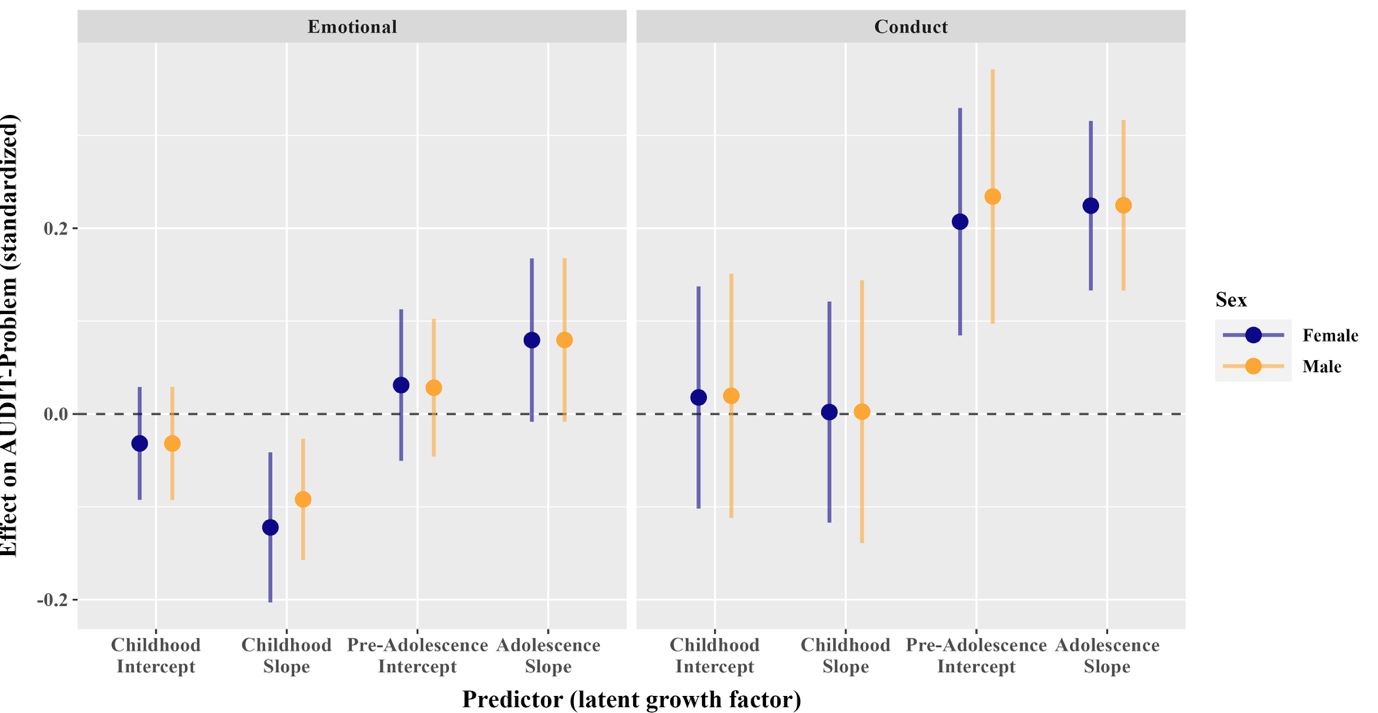
**

**Figure S1.** Multi-group analyses by sex results for latent growth factors of emotional and conduct problems predicting Alcohol Use Disorders Identification Test (AUDIT) scores (phenotypic model).

*Notes:* Standardized path coefficients and 95% confidence intervals are displayed.

**(a)**

**(b)**

**(c)**

**(d)**

**Figure S2.** Twin model results for AUDIT-Total (Alcohol Use Disorders Identification Test – Total score). Figure S2a shows results from the model with correlated A (additive genetic), C (shared environmental), and E (nonshared environmental) influences among all the predictors (the pre-adolescence intercept for conduct problems, the adolescence linear slope for conduct problems, the childhood linear slope for emotional problems) and AUDIT-Total. Figure S2b shows results from the model with direct paths from the predictors to AUDIT-Total. Figure S2c shows the modified model based on Figure S2b, where the direct path from the adolescence linear slope of conduct problems to AUDIT-Total is replaced by correlated A, C, and E paths. Figure S2d shows the same model as Figure S2c, but the C and E covariances between the adolescence linear slope of conduct problems and AUDIT-Total are dropped (this is the final model). In all models, non-significant correlations among the predictors are dropped based on results from the piecewise latent growth curve model. Bold single-headed arrows show standardized estimates of direct paths, plain single-headed arrows show A, C, E contributions to each variable. Double-headed arrows show the correlations of A, C, and E factors among the variables. 95% CIs are in parentheses.

**(a)**

**(b)**

**Figure S3.** Twin model results for AUDIT-Consumption (Alcohol Use Disorders Identification Test – Consumption). Figure S3a shows results from the model with correlated A (additive genetic), C (shared environmental), and E (nonshared environmental) influences among all the predictors (the adolescence linear slope for conduct problems, the pre-adolescence intercept for emotional problems) and AUDIT-Consumption. Figure S3b shows results from the model with direct paths from the predictors to AUDIT-Consumption (final model). Bold single-headed arrows show standardized estimates of direct paths, plain single-headed arrows show A, C, E contributions to each variable. Double-headed arrows show the correlations of A, C, and E factors among the variables. 95% CIs are in parentheses.

**(a)**

**(b)**

**(c)**

**(d)**

**Figure S4.** Twin model results for AUDIT-Problem (Alcohol Use Disorders Identification Test – Problem). Figure S4a shows results from the model with correlated A (additive genetic), C (shared environmental), and E (nonshared environmental) influences among all the predictors (the pre-adolescence intercept for conduct problems, the adolescence linear slope for conduct problems, the childhood linear slope for emotional problems) and AUDIT-Problem. Figure S4b shows results from the model with direct paths from the predictors to AUDIT-Problem. Figure S4c shows the modified model based on Figure S4b, where the direct path from the adolescence linear slope of conduct problems to AUDIT-Problem is replaced by correlated A, C, and E paths. Figure S4d shows the same model as Figure S4c, but the C and E covariances between the adolescence linear slope of conduct problems and AUDIT-Problem are dropped (this is the final model). In all models, non-significant correlations among the predictors are dropped based on results from the piecewise latent growth curve model. Bold single-headed arrows show standardized estimates of direct paths, plain single-headed arrows show A, C, E contributions to each variable. Double-headed arrows show the correlations of A, C, and E factors among the variables. 95% CIs are in parentheses.

**Figure S5.** Piecewise latent growth curve model results (phenotypic model). Model fit indices: 𝜒 ^2^ (30) = 55.67; *p* = .003; RMSEA = .01; CFI = 1.00; TLI = 1.00; SRMR = .01. Variances of latent growth factors are displayed in parentheses below the names of the latent growth factors. Standardized covariances among latent growth factors are shown with 95% confidence intervals in parentheses. All paths shown are significant at the level of *p* < .01. Other paths are non-significant (*p* > .05) and thus fixed at 0. Rectangles are measured emotional/conduct problems at ages 4, 7, 9, 11 and 16 years. Variances and covariances of residuals are not shown. Emo = emotional problems, Con = conduct problems, P = parent-report, S = self-report.

**(a) Male**


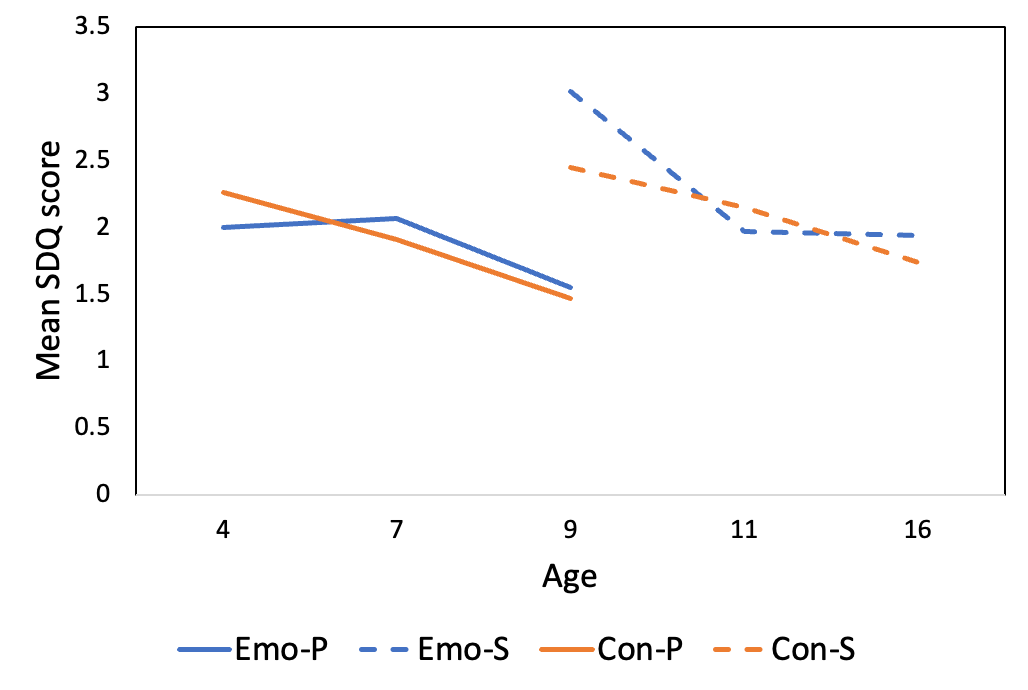


**(b) Female**


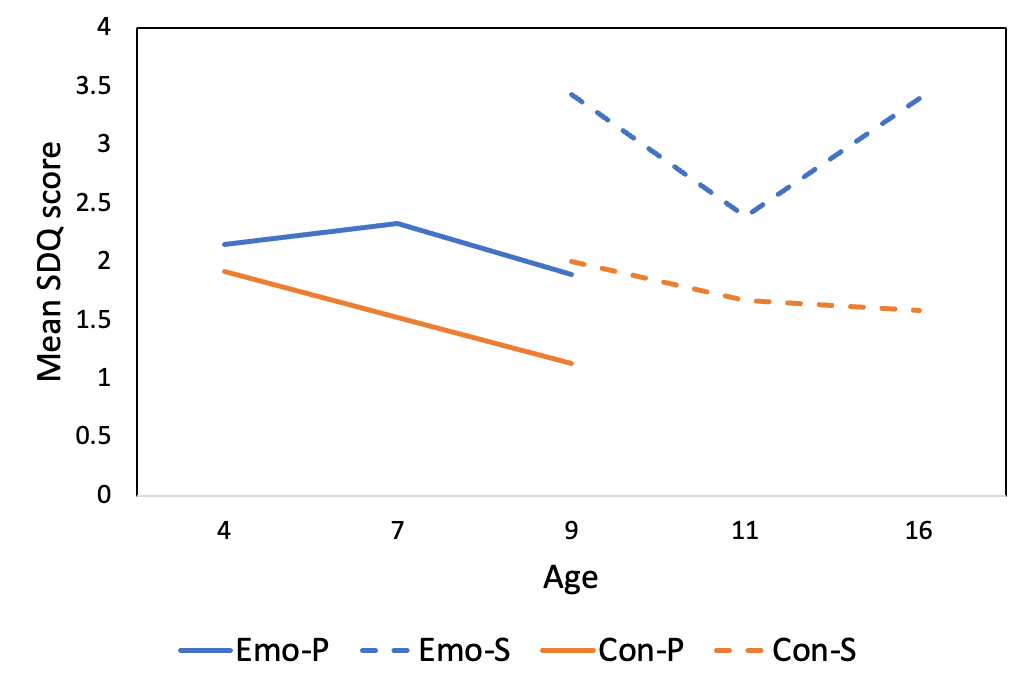


**Figure S6.** The mean trends of emotional and conduct problems across ages 4 to 16 years for males (a) and females (b). SDQ = Strengths and Difficulties Questionnaire, total score ranged from 0 to 10. Emo = emotional problems, Con = conduct problems, P = parent-report, S = self-report.

**Appendix S1.** Covariate measures.

**Family socioeconomic status.** With data from the first contact, we used a standard family socioeconomic status composite score created for the TEDS sample (e.g., Krapohl & Plomin, 2016). Indicators for family socioeconomic statuses included mother and father employment levels, mother and father educational levels, and mother's age on birth of first child. Each indicator was standardized and then averaged, then the mean score was standardized across the entire sample to create the family socioeconomic status composite.

**Ethnic origin.** Parents reported twins’ ethnicity at the first contact. White was coded as 1, non-white was coded as 0.

**Sex.** Parents report twins’ biological sexes at birth at the first contact; minor mistakes were corrected through repeated contacts.

**Relationship status.** At age 22, twins reported their relationship status. “Single”, “dating non-exclusively”, or “widowed/separated/divorced” was coded as 1. “In exclusive relationship”, “living with partner”, or “married” was coded as 0.

**Having child.** When the twins were at age 22, parents reported whether the twins had child. “Yes” was coded as 1, “no” was coded as 0.

**Living with parents.** At age 22, twins reported who were they living with. “Living with parents” was coded as 1, otherwise coded as 0.

**Educational attainment.** At age 22, twins reported their highest level of educational attainment. Categories were “no qualifications”, “General Certificate of Secondary Education (GCSE) with grades D-G, or equivalent”, “1 to 4 GCSEs with grades A-C, or equivalent”, “5 or more GCSEs with grades A-C, or equivalent”, “1 A-level pass (grades A-E)”, “2 or more A-level passes (grades A-E), or National Vocational Qualification level 3”, “1-year degree course, or equivalent (e.g., Higher National Certificate, Certificate of Higher Education)”, “2-year degree course, or equivalent (e.g., Diploma of Higher Education, Foundation degree)”, “First degree (e.g., Bachelor’s degree)”, “Master’s degree, Postgraduate Certificate in Education, or equivalent”, “Doctoral degree”. If they were still studying toward a degree, the educational attainment level was changed to the degree they were studying. The educational attainment categories were coded in order from 1 to 11.

**Employment status.** At age 22, twins reported their employment status. “Studying” or “gap year/travelling” was coded as 0. “Working”, “apprenticeship or employment training”, or “full-time parent” was coded as 1. “Unemployed” was coded as 2. The categorical variable was further dummy coded into two variables, with the first group being the reference group.

**Appendix S2**. Deviations from pre-registration.

| **Pre-registered step** | **Deviation** | **Justification** |
| --- | --- | --- |
| The study sample will include all TEDS twins whose data are available on emotional and conduct problems on at least one measurement occasion across ages 4, 7, 9 for parent-report, or across ages 9, 11, 16 for self-report, and also with data available on alcohol use at age 22. | Participants do not need to have data on age 22 alcohol use to be included in analyses. | Full information maximum likelihood handles missing data by using all available data, thus including more individuals with data on emotional and conduct problems improves estimates in the piecewise LGC model while not influencing estimates related to alcohol use. |
| Twin data will be used to decompose associations among trajectories of emotional problems, trajectories of conduct problems, and alcohol use problems at age 22 into genetic, shared and nonshared environmental factors. This research aim will be exploratory. | Only significant predictors of alcohol use behaviors were retained in the twin analyses. In addition, beyond decomposing covariances into genetic, shared and nonshared environmental factors, we tested whether associations between emotional or conduct problems and alcohol use behaviors could be explained by direct paths. | Decomposition of non-significant covariances would inevitably be underpowered, is typically unstable and is unlikely to yield interpretable results. Besides, testing whether emotional or conduct problems have direct influences on alcohol use behaviors is more useful for interpreting results obtained from phenotypic models, instead of just decomposing the covariances into genetic, shared and nonshared environmental influences for descriptive purposes. |
| Only the total score from the Alcohol Use Disorders Identification Test (AUDIT) will be used as the outcome. | Two subscales of the AUDIT: AUDIT-Consumption and AUDIT-Problem were also used as outcomes in post hoc analyses. | Although our main outcome remained the total score from AUDIT, exploring whether the effects differed for alcohol consumption versus alcohol-related problems has practical implications for targeted prevention. These analyses were also clearly mentioned as post hoc analyses in the manuscript. |

**Appendix S3.** Methods and Results for piecewise latent growth curve (LGC) models.

**Methods**

Piecewise LGC models (Flora, 2008) were used to describe developmental trajectories across childhood and adolescence for emotional and conduct problems. For each problem domain, separate intercepts were estimated in childhood (age 4, parent-report) and pre-adolescence (age 9, self-report). In addition, separate linear slopes were estimated for changes across childhood (ages 4 to 9, parent-reports), and adolescence (ages 9 to 16, self-reports). We estimated covariances between all intercepts and slopes and between the residuals of emotional and conduct problems measured at the same age. Residuals between parent- and self-reports at age 9 were also allowed to be correlated. To aid model stability and convergence, these covariances were fixed to zero if not statistically significant and the magnitude was small (ranged from .01-.16). Chi-square difference tests were also conducted to ensure that fixing these non-significant covariances did not worsen the model fit. If constraining a covariance to zero significantly worsened the model fit, the covariance was added back to the piecewise LGC model. We also tested whether the mean trends (capturing between-occasion variations) and covariance matrix (capturing individual differences) were explained by the same factor structure (i.e., an intercept and a linear slope), as is typically assumed in LGC models (see Mandys, Dolan, & Molenaar, 1994 for details). Lavaan package 0.6-12 (Rosseel, 2012) in R (R Core Team, 2022) was used for these analyses. Cluster-robust standard errors were used to account for the clustering of data within twin pairs. Full information maximum likelihood (FIML) handled missing data.

**Results**

The piecewise LGC model with a childhood intercept, a childhood linear slope, a pre-adolescence intercept, and an adolescence linear slope for both emotional and conduct problems fitted the covariance matrix well, which captured individual differences in developmental trajectories of emotional and conduct problems. However, the mean trends of emotional and conduct problems from ages 4 to 9 and from ages 9 to 16 could not be well-described by means of the intercepts and linear slopes only; an additional quadratic slope was needed to describe the mean trends. This resulted in a saturated mean structure because three factor means (i.e., intercept, linear slope, quadratic slope) were used to explain three observed means at each developmental stage of each behavior (e.g., childhood emotional problems at ages 4, 7, 9). However, the variances of the quadratic factors were fixed to 0 because they did not significantly contribute to individual differences. Therefore, only latent growth factors capturing individual differences (i.e., intercepts and linear slopes) were used to predict alcohol use behaviors. We described the mean trends in Figure S6. The variances and covariances of intercepts and linear slope factors obtained from the final piecewise LGC model are in Figure S5. Estimates from the full model before fixing non-significant covariances are in Table S5.

**References**

Flora, D. B. (2008). Specifying piecewise latent trajectory models for longitudinal data. *Structural Equation Modeling: A Multidisciplinary Journal, 15*, 513-533.

Krapohl, E., & Plomin, R. (2016). Genetic link between family socioeconomic status and children’s educational achievement estimated from genome-wide SNPs. *Molecular Psychiatry, 21*, 437-443.

Mandys, F., Dolan, C. V., & Molenaar, P. C. (1994). Two aspects of the simplex model: Goodness of fit to linear growth curve structures and the analysis of mean trends. *Journal of Educational Statistics, 19*, 201-215.

R Core Team. (2022). R: A language and environment for statistical computing. Vienna, Austria: R Foundation for Statistical Computing.

Rosseel, Y. (2012). Lavaan: An R package for structural equation modeling. *Journal of Statistical Software, 48*, 1-36.
